# Skin flora: Differences between people affected by Albinism and those with normally pigmented skin in Northern Tanzania – cross sectional study

**DOI:** 10.1186/1471-5945-12-12

**Published:** 2012-07-30

**Authors:** Samson K Kiprono, John E Masenga, Baraka M Chaula, Bernard Naafs

**Affiliations:** 1 Department of Dermatology, Regional Dermatology Training Center, Box 8332, Moshi, Tanzania; 2 Stichting Tropendermatologie, Munnekeburen, The Netherlands

**Keywords:** Skin flora, Albinism, African

## Abstract

**Background:**

Skin flora varies from one site of the body to another. Individual’s health, age and gender determine the type and the density of skin flora.

**Methods:**

A 1 cm^2^ of the skin on the sternum was rubbed with sterile cotton swab socked in 0.9% normal saline and plated on blood agar. This was cultured at 35°C. The bacteria were identified by culturing on MacConkey agar, coagulase test, catalase test and gram staining. Swabs were obtained from 66 individuals affected by albinism and 31 individuals with normal skin pigmentation. Those with normal skin were either relatives or staying with the individuals affected by albinism who were recruited for the study.

**Results:**

The mean age of the 97 recruited individuals was 30.6 (SD ± 14.9) years. The mean of the colony forming units was 1580.5 per cm^2^. Those affected by albinism had a significantly higher mean colony forming units (1680 CFU per cm^2^) as compared with 453.5 CFU per cm^2^ in those with normally pigmented skin (p = 0.023). The skin type and the severity of sun- damaged skin was significantly associated with a higher number of colony forming units (p = 0.038).

**Conclusion:**

Individuals affected by albinism have a higher number of colony forming units which is associated with sun- damaged skin.

## Background

Human skin microbiome refers to entire collection of microbes which include bacteria, archaebacteria, fungi, virus and mites [1]. The type and the number of skin microbiomes vary from one individual to another and from one site of the body to another [2,3]. Cultures and molecular analysis are used to identify the microbial inhabitants of the skin. Culture based methods are essential in isolating and identifying viable cutaneous microbes such as bacteria. However, some bacteria have factitious growth requirements and are therefore difficult to isolate [1]. Microbial genomics such as pyrosequencing is used to examine the entire complex microbial inhabitants [1]. The skin is one of the most important reservoirs of hospital acquired infections [3]. However, the skin is also regarded as a major protection barrier against invasion by various microorganisms through different mechanisms including the normal skin microbiome. There is both an intrapersonal and an interpersonal variation in skin microbiota. Bacterial colonization depends on the physiology of the site sampled with humidity and sebaceous environment influencing the type of bacteria [2]. Interpersonal variation depends on intrinsic factors such as the individual’s state of health, age and sex [4-6] and extrinsic factors such as clothing, hygiene, humidity and occupation [1,2]. There are few studies on skin flora and most of these studies involved patients and medical personnel [7,8]. The majority of studies on skin flora were done on Caucasians and to our knowledge no study has been reported in Africans with normal skin pigmentation and those affected by albinism. Those affected by albinism living in the tropics are at high risk of developing skin tumors and therefore, have to undergo many surgical interventions. It was noticed that infected wounds were encountered more often in those affected by albinism than those who were not. Identification, qualification and quantification of skin flora may help in the preoperative antiseptic measures in these patients. Therefore, the objective of this study was to determine and to compare the normal aerobic bacterial skin flora in Africans affected with Albinism with those with normally pigmented skin.

## Materials and methods

### Setting and sample

This study was conducted in eight of the Regional Dermatology Training Center Albino outreach clinics. Most of them served farmers. The study was approved by the Tumaini University Research and Ethics committee and conducted according to the guidelines by the committee. Sixty-six individuals affected with Albinism and 31 individuals with normally pigmented skin (ratio of 2:1) were recruited for the study. Those with ulceration, abscesses or other skin diseases were excluded.

### Procedure

A structured questionnaire was used to collect data on the demographics and possible factors influencing the skin flora. The pre-sternal area 4 cm below the sterno-clavicular joint, which is exposed and easily accessible, was used as site for sample collection. The sample collection area was mapped using an aluminum squared rim with a total free area of 1 cm^2^. The squared rims were cleaned with 70% alcohol for three minutes and left to dry before being reused. The samples were collected using a sterile cotton swab, moistened with sterile 0.9% sodium chloride. A five stroke swabbing was done with a clockwise movement with a pressure similar to that of a pencil eraser. All samples were collected by the same individual. The swab was placed in a tripticase soy broth transport media and plated within 4 hours on blood agar. The blood agar plates were incubated at 35 °C and examined at 24- and 48 hours and the bacterial colonies were identified using standard procedures and recorded as colony forming unites (CFU) per cm^2^. CFU/cm^2^ is used as a measure of the number of microorganisms present on the surface of the sample. Culture on MacConkey agar, coagulase test, catalase test and gram stain were used to classify aerobic bacteria and identify staphylococcus, streptococcus, gram-negative cocci and bacilli, which may be potentially pathogenic. Anaerobes were not looked for.

### Data analysis

A statistical package for social scientists (SPSS Chicago Inc.) software was used for data analysis. A descriptive analysis was done to characterize the sample, while chi-square, Fisher’s Exact test and t-test were used to determine the association between variables depending on the sample size or for comparing the means. Sun damage was classified as absent (none and mild erythema) and present (moderate and severe erythema). Assessment of erythema in Black Africans is difficult, especially distinguishing between no erythema and mild erythema. Therefore, both were grouped into the group without sun damage. The cut-off for considering the CFU/cm^2^ as low or high was set at 600 CFU/cm^2^ similar to that reported in other studies [4,5]. All statistical analysis were 2-tailed and considered significant at p < 0.05.

## Results

A total of 97 individuals of whom 50 (51.5%) were females giving a male to female ratio of 1:1.1 were included in this study. Their mean age was 30.6 years SD ± 14.9 and a median of 30 years (range 5-77 years). Seventy-four (76.2%) individuals had bathed not more than12 hours earlier and 20 (20.6%) individuals had used medicated soaps. The mean age of Albinos was 28.6 years (SD ± 13.8) and was 34.8 years (SD ± 16.5) for those with normally pigmented skin. This difference was not statistically significant (p = 0.057). Sun damage was found to be statistically different between the two groups as shown in Table 1.

**Table 1:** A comparison of characteristics of Albinos and those with pigmented skin

	**Albino**	**Pigmented**	**P value**
	**N = 66**	**N = 31**	
**Age in years (mean)**	28.6	34.8	.057^†^
**Gender (Female)**	33(50)	17(54.8)	.662*
**Bathing within 12 hours**	49(74.2)	25(80.5)	.523*
**Medicated soap**	12(18.2)	8(25.8)	.412*
**Daily bathing**	54(81.9)	21(67.7)	.843*
**Changing clothes daily**	38(57.6)	20(64.5)	.518*
**Pustules in previous month**	6(9.1)	1(3.2)	.424^∞^
**Skin sun damage**	34(51.5)	0	.000^∞^

*Chi-square test.

†Independent samples *t* test.

^∞^Fisher’s Exact test.

The mean CFU for the combined population was 1580.5 CFU/cm^2^ (SD ± 30294). The mean CFU in individuals with normally pigmented skin was 453.5 CFU/cm^2^ (SD ± 1795) and 1680 CFU/cm^2^ (SD ± 35596 in those affected by albinism. This difference was statistically significant (p = 0.023). A bivariate analysis showed that skin type and sun- damaged skin was significantly (p = 0.038) associated with high number of colony forming units (more than 600 CFU/cm^2^) as shown in Table 2. Sun damage was still a significant factor (p = 0.046) for high number of colony forming units when analysis is done for albino group.

**Table 2:** Bivariate analysis of factors associated with High CFU (>600 CFU/cm^2^)

**Characteristic**	**Total**	**High CFU**	**p value**
**Age**			
16 years and younger	19(19.6%)	6(31.6%)	.600*
Older than 16 years	78(80.4%)	25(32.1%)	
**Type of skin**			
Albino	66(68%)	27(40.9%)	.006^†^
Pigmented skin	31(32%)	4(12.9%)	
**Gender**			
Male	47(48.5%)	15(31.9%)	.593*
Female	50(51.5%)	16(32.0%)	
**Type of employment**			
Medical	2(2.1%)	1(50.0%)	.539^†^
Non-medical	95(97.8%)	30(31.6%)	
**Bathing within 12hours**			
Yes	70(72.2%)	22(31.4%)	.520*
No	27(27.8%)	9(33.3%)	
**Type of soap**			
Medicated	20(20.6%)	5(25.0%)	.322*
Non-medicated	77(79.4%)	26(33.8%)	
**Sun damage**			
Absent	63(65.0%)	14(22.2%)	.005*
Present	34(35.0%)	17(50%)	

*Chi-square test.

†Fisher’s Exact test.

The cultures of the 97 samples showed Coagulase negative staphylococcus in 94.8%, whereas Coagulase positive staphylococcus was found in 13.4% of the samples. Gram negative bacteria (28.9%), Bacillus spp (22.7%), α-hemolytic streptococcus (5.2%) and β-hemolytic streptococcus (3.1%) were also isolated.

## Discussion

The characteristics of the study groups were similar except for the sun damage. The mean CFU/cm^2^ for the African population in this study was significantly higher than that in most reported studies. Larson et al [5] reported a significantly higher number of colony forming units among African Americans. The variation may be because of external factors like humidity, weather conditions, type of clothing and the level of hygiene, which has been reported to influence bacterial skin colonization [9].

The mean number of CFU/cm^2^ in individuals affected with albinism was statistically higher than those in individuals with normally pigmented skin. The differences in the study populations in recent studies [4,5] make it difficult to compare the results with the results of this study. For instance, the mean number of CFU/cm^2^ in normally pigmented skin (453.5 CFU/cm^2^) was higher than the 320 CFU/cm^2^ among patients in Thailand [4], but the median was similar.

Leyden et al [8] in USA reported that the density of the micro-flora on the dry areas of the skin including the sternum ranged from 10^3^-10^4^ CFU/cm^2^ ,which is similar to our findings in the normally pigmented skin, but lower than that in individuals affected by albinism. Sun damage was significantly associated with a higher number of colony forming units. Ultra-violet (UV) light is known to be bactericidal [10] and therefore, it is expected that bacterial colonization would be lower in regions close to the equator. The bactericidal effect may be counteracted by the immunosuppressive effect of UV light on the local and systemic immunity [11]. Sun- damaged skin loses the barrier function increasing the susceptibility to colonization by bacteria. However, neither the skin barrier function nor the antimicrobial peptides (AMPs) were assessed in this study. Sun- damaged skin is often slightly thickened and not entirely smooth providing areas of invaginations that act as reservoirs where bacteria flourish and from which the skin surface is re-colonized [1].

Bathing and the use of antimicrobial soap had no statistical influence on the number and the density of CFU/cm^2^. Larson et al [12] reported that hand washing and the use of antimicrobial soaps was associated with a slight reduction in the number of CFU/cm^2^, but that no individual was free of organisms. Single use of antimicrobial soaps, duration of bathing and the frequency of bathing may influence re-colonization [13]. Moreover, the use of antimicrobial soaps may even damage the normal barrier function of the skin.

Staphylococcus was the commonest microorganism isolated, which concurs with other studies, which reported staphylococcus to be present in more than 90% of the samples [4,5]. However, gram- negative bacteria were present in more (28.5%) samples than that reported by Larson et al [4] among inpatient (9.2%) and outpatients (17.2%).

## Conclusion

In Africa, individuals affected by albinism have a higher density of skin micro-flora than the normally pigmented African population. Staphylococcus is the most common organism isolated. However, there is relatively more colonization with gram- negative bacteria. The impact of these results on bacterial skin infections and surgical site infection needs further investigation.

## Competing interests

The authors declare that they have no competing interests.

## Authors’ contribution

SK and BC made substantial contribution in the concept and the study design, collection, analysis and interpretation of the data and drafting of the manuscript, whereas JM and BN contributed in the concept and the design, data interpretation and critical revision of the manuscript with regards to the intellectual content. All authors read and approved the final manuscript.

## Pre-publication history

The pre-publication history for this paper can be accessed here:

http://www.biomedcentral.com/1471-5945/12/12/prepub

## References

[B1] Kong HH Skin microbiome: genomics-based insights into the diversity and role of skin microbes Trends Mol Med 2011 17 6 320 328 10.1016/j.molmed.2011.01.013 21376666 PMC3115422

[B2] Grice EA Segre JA The skin microbiome Nature Rev Microbiol 2011 9 244 253 10.1038/nrmicro2537 21407241 PMC3535073

[B3] Noble WC The skin microflora and microbial diseases 2004 Philadelphia PA, Cambridge University Press

[B4] Thamlikitkul V Santiprasitkul S Suntanondra L Skin flora of patients in Thailand Am J Infect Control 2003 31 80 84 10.1067/mic.2003.64 12665740

[B5] Larson EL Cronquist BA Whittier S Lai L Lyle CT Latta PD Differences in skin flora between inpatients and chronically ill outpatients Heart Lung 2000 29 298 305 10.1067/mhl.2000.108324 10900068

[B6] Marples RR Sex, constancy, and skin bacteria Arch Dermatol Res 1982 272 317 320 10.1007/BF00509062 7165340

[B7] Larson EL Hughes CAN Pyrek JD Sparks SM Cagatay EU Bartkus JM Changes in bacterial flora associated with skin damage on hands of health care personnel Am J Infect Control 1998 26 513 521 10.1016/S0196-6553(98)70025-2 9795681

[B8] Leyden JJ McGinley KJ Nordstrom KM Webster GF Skin microflora J Invest Dermatol 1987 88 65s 72s 10.1111/1523-1747.ep12468965 3102625

[B9] Fierer N Lauber CL Zhou N McDonald D Costello EK Knight R Forensic identification using skin bacterial communities Proc Natl Acad Sci USA 2010 107 6477 6481 10.1073/pnas.1000162107 20231444 PMC2852011

[B10] Faergemann J Larko O The effect of UV-light on human skin microorganisms Acta Derm Venereol 1987 67 69 72 2436418

[B11] Hanneman KK Cooper KD Baron ED Ultraviolet immunosuppression: mechanisms and consequences Dermatol Clin 2006 24 1 19 25 10.1016/j.det.2005.08.003 16311164

[B12] Larson EL Gomes-Duarte C Lee LV Della-Latta P Kain DJ Keswick BH Microflora of hands of homemakers Am J Infect Control 2003 31 72 9 10.1067/mic.2003.33 12665739

[B13] Borges LF Silva BL Filho PP Gerais M Hand washing: Changes in the skin flora Am J Infect Control 2007 35 417 20 10.1016/j.ajic.2006.07.012 17660014

